# Spatiotemporal patterns of clinical bovine dermatophilosis in Zimbabwe 1995–2014

**DOI:** 10.4102/ojvr.v84i1.1386

**Published:** 2017-06-27

**Authors:** Felistas Ndhlovu, Daud N. Ndhlovu, Sylvester M. Chikerema, Mhosisi Masocha, Mudavanhu Nyagura, Davies M. Pfukenyi

**Affiliations:** 1 Division of Veterinary Services, Department of Livestock and Veterinary Services, Zimbabwe; 2 Department of Clinical Veterinary Studies, University of Zimbabwe, Zimbabwe; 3 Department of Geography and Environmental Science, University of Zimbabwe, Zimbabwe; 4 Department of Pre-Clinical Veterinary Studies, University of Zimbabwe, Zimbabwe

## Abstract

A retrospective study of clinical bovine dermatophilosis outbreaks and cases for the period 1995–2014 was conducted, using data obtained from the Division of Veterinary Services (DVS). A total of 3856 outbreaks and 26 659 cases of dermatophilosis were reported countrywide during this period. The post rainy season accounted for 37.9% of the outbreaks followed by the rainy season (26.7%), cold dry season (22.1%) and the hot dry season (13.2%). A retrospective space–time scan statistic in SaTScan™ was used to detect clusters. From this study, it was evident that dermatophilosis was spreading from the north-west of Zimbabwe through the central to the north-east during the period 2010–2014. Five clusters were identified mainly in the central and north-western regions of Zimbabwe. The primary cluster was centred at Ungwe, Gokwe district in Midlands; the second, third, fourth and fifth likely clusters were centred at Bonga (Mashonaland Central), ARDA (Mashonaland West), Nsenga (Matabeleland North) and Zanda in Gokwe, respectively. The findings of this study suggest the continued spread of dermatophilosis across the country; as such the Department of Livestock and Veterinary Services are advised to develop measures aimed at managing this spread such as dipping, quarantine, movement control and raising farmer awareness.

## Introduction

Dermatophilosis is an economically important disease of livestock caused by *Dermatophilus congolensis*, a Gram-positive actinomycete that produces motile zoospores which invade the skin (Hadrill & Walker 1996; Samson et al. 2010). Although it has a worldwide distribution, the disease is reported mainly in African countries (Awad, Nadra-Elwgoud & El-Sayed 2008). The disease is of particular importance in the tropics and sub-tropics where it causes substantial losses (Woldemeskel & Taye 2002). Dermatophilosis has been reported in cattle, camels, buffaloes, donkeys, cats, dogs, wildlife and man (Awad et al. 2008; Amor et al. 2011) but is more severe in ruminants (Dalis et al. 2009; Woldemeskel & Mersha 2010).

Although tick-free animals are susceptible to infection and clinical disease, the disease is more severe in animals infested with *Amblyomma variegatum* ticks (Ambrose 1996). As such, occurrence and spread of dermatophilosis in cattle has been associated with *A. variegatum* (Ahoussou et al. 2010). Walker (1996) postulated that an immunosuppressive agent secreted in the tick’s saliva or waste metabolites led to the development of dermatophilosis. Clinically, bovine dermatophilosis presents as an exudative dermatitis with lesions that can be localised or generalised (Koney et al. 1996). The lesions are distributed mainly in the inguinal area, scrotum and front legs in males; in females, they are mainly around the udder and external genitalia and on the back in both sexes (Chatikobo et al. 2001; Dalis et al. 2009). This distribution has been reported to adversely affect mating and fertility. A carrier state has been reported in cattle by Stewart (1972), such carrier cattle do not present with observable clinical signs and were postulated to be the chief means of survival for *D. congolensis*.

Economically, bovine dermatophilosis is important due to morbidity and mortality, damage to hides and its effect on draught animal power (Samui & Hugh-Jones 1990). Hide condemnations are an important cause of economic loss for the leather industry and in countries where cattle hides are processed for human consumption (Cadmus & Adesikan 2009). In addition, introduction of exotic breeds to improve meat and milk production has been frustrated in other parts of Africa (Koney 1996) by this disease. The disease is also of public health significance as it can be transmitted to humans (Awad et al. 2008; Amor et al. 2011).

In Zimbabwe, bovine dermatophilosis is a notifiable disease with two statutory instruments (SI) developed in 2010 for its management and control. Statutory Instrument 166 of 2010 (Animal Health [Dermatophilosis Areas] Order 2010a) defines certain districts in the country as dermatophilosis prescribed areas. SI 167 of 2010 (Animal Health [Dermatophilosis] Regulations 2010b) regulates what should be done by farmers and authorised persons for the control of dermatophilosis. The SI place a legal obligation on the farmer or anyone to report occurrences of suspected bovine dermatophilosis to the nearest veterinary offices. Persistent droughts and the land resettlement programme have been postulated as the reasons for the spread (Chatikobo et al. 2009), while Ndhlovu and Masika (2013) cited the decrease in government support to the smallholder farmer dipping programme and the economic challenges faced by the country at the time as further contributing factors.

Studies on bovine dermatophilosis in Zimbabwe have focused on tick control, prevalence and distribution of the disease in certain areas of the country (Chatikobo et al. 2001, 2004, 2009; Ndhlovu & Masika 2013, 2015). However, little has been done on the spatiotemporal characteristics of the disease. Various spatial statistical analysis techniques and software are available. These techniques use spatially referenced data for the development and analysis of spatial models (Li, Guo & Elkan 2011).

SaTScan™ (2016) is a free downloadable software that can be used to analyse spatial, temporal and space–time data using the spatial, temporal and space–time scan statistics within the software SaTScan™ version 9.4.4 (Software for the Spatial and Space-Time Scan Statistics 2016). The scan tests detect the location and test the significance of clusters using a search or scanning window (Heres, Brus & Hagenaars 2008). The scanning window moves across space and/or time; for each location and size of the window, the number of observed and expected cases are counted (Kulldorf et al. 2005). Scan statistics can be used to detect and test the presence of purely spatial, purely temporal and space–time clusters (Szonyi, Wade & Mohammed 2010). The detection and scanning is performed, briefly, as follows: for purely spatial analysis a circular or oval scanning window is used and in space–time analysis a cylindrical window is utilised, the base of this cylinder represents the geographic search area while the height represents the time frame (Szonyi et al. 2010; Wolff et al. 2014). The observed and expected number of cases inside the search window is compared with those outside the window, and a likelihood ratio test is performed to confirm if the risk of disease inside the window is the same as in the outside (Szonyi et al. 2010). The window with the highest likelihood ratio is considered the most likely cluster and a *p*-value is assigned to it.

The current study was undertaken to determine the spatial and temporal trends, associated with the occurrence of dermatophilosis in Zimbabwe, as well as to identify possible clustering of the disease. This study used passively collected data on clinical dermatophilosis. Findings from this study will assist in the formulation of targeted control strategies aimed at preventing further spread of the disease with the ultimate goal of improving smallholder livestock production and productivity.

## Materials and methods

### Study population and data collection

A retrospective study of clinical bovine dermatophilosis outbreaks and cases for the period 1995–2014 was conducted. Dermatophilosis outbreaks and cases were identified through the Division of Veterinary Services (DVS) disease reporting system. This system is made up of a computerised database created using disease and epidemiology reports submitted from 60 districts and 8 provincial veterinary offices of the country. Data in the system are collected through an animal disease surveillance system that combines both passive and active elements. The passive component is based on regular disease reporting by farmers with subsequent follow-up by veterinary officers (VO), this system can suffer from under reporting. Dermatophilosis is notifiable in Zimbabwe (Statutory Instrument 166 of 2010 [Animal Health {Dermatophilosis Areas} Order 2010a]; SI 167 of 2010 [Animal Health {Dermatophilosis} Regulations 2010b]), as such under reporting is mitigated as reporting the occurrence of the disease is mandatory. This system is augmented through monthly visits conducted by veterinarians, animal health inspector and veterinary extension assistants (VEAs) to dip tanks and farms. While these visits are not specifically for the detection of bovine dermatophilosis, they increase the likelihood that the disease will be detected and appropriate follow-ups made. The data on disease occurrences that are reported and investigated are initially captured in a disease investigation form (DIF). The DIF has a number of sections that capture biographical data of the farmer reporting such as, the geographical location, number of cases, herd size, clinical signs and whether it is an initial or a follow-up report. The section for diagnosis is divided into two parts, the first is completed by an officer other than a veterinarian and in this section the tentative diagnosis is captured. The final section is for the provisional/definitive diagnosis, this part is filled in by the veterinarian upon referral of the report by a non-veterinarian and also in the event that the disease occurrence is reported directly to the veterinary officer. Disease reports were generated when VO, animal health inspectors (AHIs) and/or VEAs investigated disease occurrences that were reported by livestock owners. Diagnoses were made by VOs, AHIs and VEAs with reports from AHIs and VEAs further verified by the VOs for accuracy. Diagnoses were based on the presenting clinical signs and the provisional diagnosis was verified based on adherence to a prescribed case definition for bovine dermatophilosis, geographical area, and season. Cases presenting with a case definition that satisfied any one of the following clinical signs (lesions): small paint brush-like clumping of hair, multiple circumscribed scabs over 1 cm in diameter and confluent progressive lesions as described by Hadrill and Walker (1996), were considered clinically to be bovine dermatophilosis cases. The officers making the diagnoses were trained in animal health and disease recognition and possessed qualifications ranging from diploma in animal health to veterinary science degrees. Data on new cases were used to avoid cases that were reported as follow-ups and chronic cases; this was done by filtering the data set to include only records that were flagged as new and excluding all records that were flagged as follow-ups in the database.

### Data manipulation: Geo-referencing and epidemiological units

Disease data were captured in Microsoft Excel^®^ (2013); these data were geo-referenced using the coordinate system (latitude and longitude). In the event that a farm or dip tank (epi-unit) had different geo-references (latitudes and longitudes) for the number of times reports were made from that location, the coordinates were corrected for each by using the list of dip tanks and farms with Global Position System (GPS)-derived coordinates that had been collected by the DVS since 2010. Data were checked for multiple entries; these occurred when the same case and the related information was entered more than once, such additional entries were deleted. Records that had zero cases and deaths were deleted. Outbreaks and cases were identified to an epidemiological unit (epiunit) which was either a dip tank or a farm. An outbreak was defined as the occurrence of a disease event at a point in time and place that complied with the case definition of bovine dermatophilosis, and these data were used for temporal analysis. Cases were defined as the total number of cattle at a point in time and place, presenting with clinical signs that complied with the case definition of bovine dermatophilosis. The individual clinical cases data were used in the spatial analysis using SaTScan™ version 9.4.4 (Software for the Spatial and Space–Time Scan Statistics 2016) and in temporal analysis.

### Temporal analysis

Data were analysed using the Software Package for Social Scientists (SPSS) (International Business Machines^®^ SPSS^®^ version 21 2012). Clinical dermatophilosis cases and outbreaks were tested for normality using the Kolmogorov–Smirnov Test, a *p*-value of < 0.05 indicated non-normality of the distribution. Cases and outbreaks were further pooled according to seasons of the year. Seasons of the year were defined according to Chikerema et al. (2012) as follows: rainy (December–February), post rainy (March–May), cold dry (June–August) and hot dry (September–November). This definition of seasons is a variation to that described in the *Climate Handbook of Zimbabwe* Meteorological Services of Zimbabwe (1981). The handbook defines the seasons as follows: main rainy season; mid-November to mid-March, post rainy season; mid-March to mid-May, cool season; mid-May to August and hot season; September to mid-November. According to the *Climate Handbook of Zimbabwe* (Meteorological Services of Zimbabwe 1981), the mean temperature was reported to be highest, at 32 °C, in October the mid-point of the hot dry season, 12.8 °C – 18.6 °C in July at the mid-point of the cold dry season and a low of 9 °C in January the mid-point of the rainy season. Average annual rainfall for Zimbabwe was reported to be 675 mm, of this; the least rainfall, that is, ≤ 0.2%, was received in October and April the mid-points of the hot dry and post rainy seasons, respectively, while the most average rainfall of ≤ 100 mm was received during rainy season, and ≤ 10 mm of rainfall received in the cool dry season (Meteorological Services of Zimbabwe 1981). Descriptive statistics, that is, proportions of pooled seasonal cases and outbreaks, were computed. The relationship between the number of clinical cases and outbreaks, as dependent variables, and the categories – month, season and year – as independent variables, were evaluated using the Kruskal–Wallis one-way ANOVA for independent samples, and the median test both non-parametric tests. Post-hoc multiple pairwise comparison tests were performed using the same test in SPSS^®^ (International Business Machines^®^ SPSS^®^ version 21 2012). A *p*-value of < 0.05 was considered as the significance level. Separate box-plots for the pooled dermatophilosis clinical outbreaks and cases, according to month, season and year, were constructed.

### Space–time analysis

To detect and test clusters, the scan test in SaTScan™ version 9.4.4 (Software for the Spatial and Space–Time Scan Statistics 2016), a statistical software, was used, and the *p* < 0.05 was considered as significant. The null hypothesis was that the number of cases were randomly distributed throughout the dip tanks and farms and that the time frames of occurrence of cases were similar, the alternative hypothesis was that cases were clustered. The variables used for the analysis were the location, coordinates, dates and number of cases. Retrospective space–time analysis was used, using the space–time permutation probability model, scanning for areas with high rates. The study period was from 01 January 1995 to 31 December 2014 and time precision was a month. The maximum spatial cluster size was set at 25% of the population at risk while the maximum temporal cluster size was at 20% of the study period and time aggregation was set at 3 months. These parameters which were lower than those recommended (Kulldorf 2015) were selected to make the scan more specific. The scan test detects and tests for clusters in the following manner, the observed and expected number of cases inside the search window are compared with those outside the window and a likelihood ratio test is computed to test if the risk of disease inside the window is the same as outside (Szonyi et al. 2010). The window with the highest likelihood ratio is considered the most likely cluster and a *p*-value is assigned to it. The likelihood ratio test is computed by comparing the likelihood or probability of occurrence of an event or parameter under the alternate and null hypothesis (Dohoo, Martin & Stryhn 2003). The likelihood ratio test was run with 999 Monte Carlo replications. Monte Carlo is a type of simulation that relies on repeated random sampling and statistical analysis to compute the results (Raychaudhuri 2008); these statistical analyses are performed using a computer (Munfrom et al. 2011). The output of the analysis was projected onto Quantum Geographical Information System (QGIS) version 2.14.5-Las Palmas de G.C. (2017) an open source geographic information management system software.

## Results

### Temporal distribution of dermatophilosis outbreaks

**FIGURE 1:**
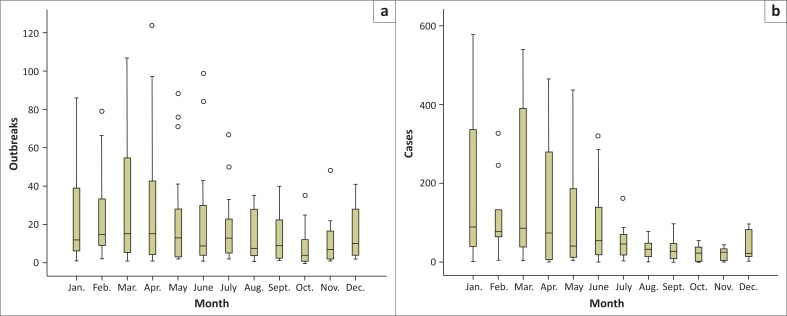
Monthly distribution of dermatophilosis (a) outbreaks and (b) cases for the period 1995–2014.

**FIGURE 2:**
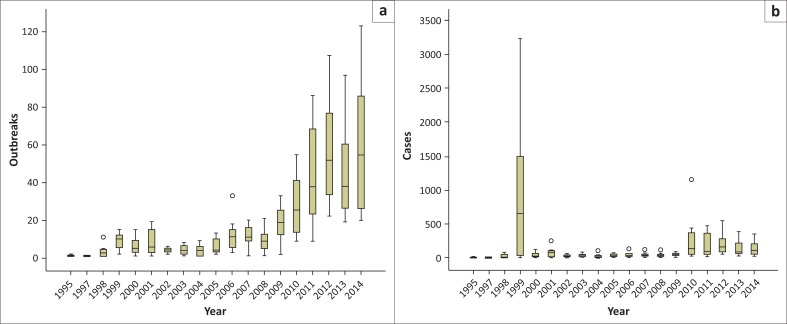
Annual distribution of dermatophilosis (a) outbreaks and (b) cases for the period 1995–2014.

**FIGURE 3:**
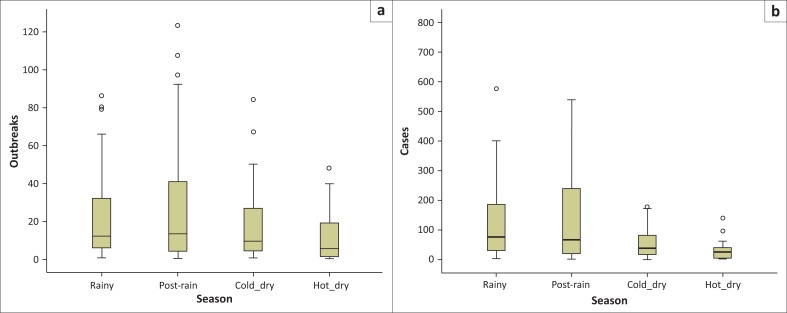
Seasonal distribution of dermatophilosis (a) outbreaks and (b) cases for the period 1995–2014.

A total of 3856 outbreaks with 26 659 cases of clinical bovine dermatophilosis were reported countrywide during the period 1995–2014. The temporal distribution of outbreaks and cases reported for the period are indicated as box-plots in Figures 1, 2 and 3. The post rainy season accounted for 37.91% of the outbreaks followed by; the rainy season (26.73%), cold dry season (22.14%) and the hot dry season (13.22%). Dermatophilosis was reported throughout the year with most outbreaks occurring in the first third of year (Figure 1) with the minimum and maximum monthly median outbreaks for the period being 4 and 15 during October and the period February–April respectively. The study period 1995–2008 was characterised by fewer outbreaks (minimum and maximum median outbreaks of 1 and 11.5 in 1997 and 2006 respectively) than the period 2009–2014 which had comparably more outbreaks (minimum and maximum median outbreaks of 19.5 and 54.5 during 2009 and 2014 respectively) (Figure 2). The number of cases across the years were characterised by a notable spike in 1999 (median 644) and more cases for the period 2010–2014. The post rainy season accounted for most of the outbreaks and cases followed by the rainy season (Figure 3). The distribution of median outbreaks across seasons was significantly different (*p* = 0.013). Multiple pairwise comparisons showed that outbreaks during the hot dry season were significantly different (*p* = 0.022) from those during the post rainy season, a significant difference (*p* = 0.030) was also observed between the rainy and hot dry season. The median number of cases differed significantly across seasons (*p* = 0.0001). The median number of cases during the hot dry season differed significantly from the number during the rainy season (*p* = 0.001), the post rainy season (*p* = 0.0001) and cold dry season (*p* = 0.044).

### Clusters detected

**FIGURE 4:**
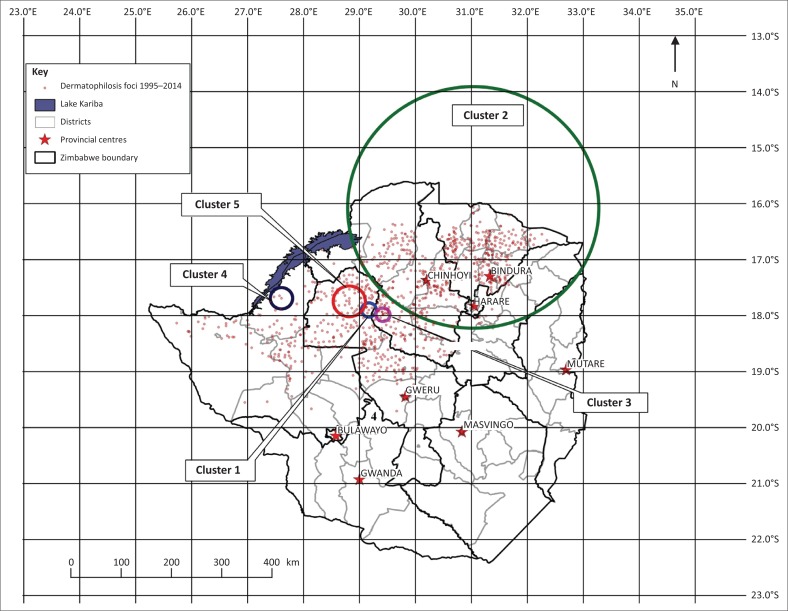
Likely clusters of clinical dermatophilosis cases in Zimbabwe for the period 1995–2014.

Five likely clusters of varying sizes were detected using SaTScan™ (2016); the locations of the clusters are shown in Figure 4. Details of the clusters are summarised in Table 1. The clusters were located mainly in the north-west parts of Zimbabwe and were of varying spatial and temporal sizes.

**TABLE 1:** Likely clusters from a space–time scan statistic (space–time permutation model) of clinical dermatophilosis.

Cluster number	Cluster centre	Radius (km)	Cluster period		Number of locations	Number of cases		Likelihood ratio	*p*
			From	To		Observed	Expected		
1	Ungwe	14.0	1999/1/1	1999/9/30	7	5580	2281.7	1927.9	0.00001
2	Bonga	239.7	2011/1/1	2014/12/31	458	3495	1317	1333.1	0.00001
3	ARDA	13.3	1999/1/1	1999/6/30	12	3800	1522	1305.5	0.00001
4	Nsenga	21.2	2010/1/1	2010/3/31	4	789	72	1179.6	0.00001
5	Zanda	30.5	2001/1/1	2001/3/31	26	576	34	1091.8	0.00001

## Ethical consideration

The Division of Veterinary Services, Zimbabwe, consented to the use of the data. Farmers handle the animals and treat them with due care taken for their welfare.

## Discussion

Findings of this study were limited by the type of data used, which were collected passively. Such data make it difficult to identify instances of missing data because of either underreporting or failure by staff to capture the data electronically. However, these findings can be used as baseline data, by animal health authorities and researchers to develop targeted disease control strategies and research on putative risk factors for the occurrence of dermatophilosis.

The disease was reported for all the years from 1995 to 2014 although at different levels. The findings of this study suggest an increase in the number of outbreaks and cases starting from 1999 onwards as shown by the increase in median outbreaks and cases. The first increase in 1999–2000 could have been due to the effect that the start of the land reform programme had on dipping and other animal health related activities. During this period as stockowners moved their cattle from one area to another, dipping schedules could have been missed or the dipping services not yet well established at the new properties, leading to increased outbreaks of tick-borne and tick associated diseases. Previously, Lawrence, Foggin and Norval (1980) reported that the disruptions in dipping and other animal health activities during the war of independence led to increased livestock deaths due to diseases. The second peak around the period 2006–2007 could have been associated with the start of the economic challenges that peaked in 2008. This also could have led to disruptions in animal health control activities. The general increase in outbreaks throughout the years and particularly around 2014 could be due to the fact that the disease had attained pandemic proportions as was previously predicted by Chatikobo et al. (2009). Increased reporting of the disease by officers may also have contributed to gradual increase in outbreaks reported. The data used in the study was based on a passive surveillance system, as such, there is a possibility of under- or over-reporting due to misdiagnosis; not all farmers report disease occurrences to veterinary authorities and they usually treat their livestock without such consultation, the disease is notifiable in Zimbabwe, it is mandatory for farmers to report it to national authorities. Notable were the low number of outbreaks and cases characterising the period 1995–1998. The computerised information management system for capturing disease data was established in 1995, as such it is possible that that reports on dermatophilosis and other diseases were not captured in the system as officers were not yet used to the system resulting in underreporting during the early years of the system being established.

The current study suggested that dermatophilosis outbreaks occurred throughout the year and most outbreaks occurred during the rainy and post rainy seasons. Due to the established association between *A. variegatum* and dermatophilosis (Ahoussou et al. 2010; Stachurski, Zoungrana & Konkobo 2010), the seasonality of the disease could be explained by the seasonality of the tick infestation burdens. Unganai (1996) reported that the main precipitation months are December to February although precipitation can span the months of October to April depending on the region of the country. Norval (1983) and Walker et al. (2003) reported that *A. variegatum* was present throughout the year although heavier infestations occurred during the warmer months (September–May) than in the cooler months (June–September). This temporal distribution implies that control of the disease should not only be limited to the rainy and post rainy season but throughout the year. Given the chronic nature of the disease, it is likely that some of the cases reported during other seasons were not new cases/infections; in the data collection, follow-up cases were excluded so this proportion might be minimal. It is postulated in this study, that farmers who report a dermatophilosis occurrence and are given advice do not usually return to give the same report later.

Disease clusters occur when cases of the disease occur in closer succession and in a smaller area than would be expected by chance alone. Such clusters occur in space and time (Wolff et al. 2014). Five clusters were identified mainly in the central and north-western regions of Zimbabwe. The primary cluster (cluster 1) which occurred between 01 January 1999 and 31 December 1999 was located in Ungwe, Gokwe district in the Midlands province, bordering with Kadoma in Mashonaland West province. The space–time cluster corroborates reports by Chatikobo et al. (2009) who reported that outbreaks of dermatophilosis occurred in this area around 1999. The explanation given by the farmers who were interviewed was that cattle in the area had not been dipped for the past five-six months resulting in outbreaks of the disease (Chatikobo et al 2009). The second cluster (cluster 2) was centred at Bonga dip tank, Mbire district in Mashonaland Central province. Cluster 2 had a radius of 239.7 km and included the Mashonaland West, Mashonaland Central and Mashonaland East provinces with a cluster period from 01 January 2011 to 31 December 2014. The Bonga cluster had the largest radius and longest period spanning 4 years. This cluster could have resulted from inter district and inter provincial movement of *A. variegatum* tick infested, infected or carrier cattle from the south-western provinces going eastwards. Most of the cattle markets are lucrative in the north-east parts of Zimbabwe. The third cluster was centred at ARDA Gokwe North district in the Midlands province, this cluster had a radius of 13.3 km and was clustered within the period 01 January 1999 to 30 March 1999. Location of cluster 3 was consistent with reports that dermatophilosis has initially and consistently been reported from the north-west of Zimbabwe, this as a result of its association with *A. variegatum* that is prevalent in this part of Zimbabwe (Chatikobo et al. 2001; Norval 1983; Walker et al. 2003). Nsenga farm in Binga district, Matabeleland North province was the centre for cluster 4, spanning a period from 01 January 2010 to 31 March 2010. According to Chatikobo et al. (2009) dermatophilosis spread from this north-west area going north-eastwards. The fifth cluster was centred at Zanda in Gokwe district. Clusters 1, 3 and 5 were located in the part of the country where dermatophilosis has been consistently reported from (Chatikobo et al. 2009; Ndhlovu & Masika 2015).

An examination of the cluster periods suggests that dermatophilosis, according to the current study period, might have started from clusters 1 and 3 (01 January 1999), then spread to clusters 5 and 4 and finally cluster 2 (31 December 2014). The spatial and temporal proximities of clusters 1, 3 and 5 suggest that it could be possible that movement of disease from one area to the other was due to an increase in cattle movements within and between districts; such movements could be for sale purposes or for socially related activities. Furthermore, these three clusters were associated with the period when the land reform was at its peak; this programme was associated with movements of livestock from one district to another district where people had been allocated land for resettlement. The space–time analysis is in agreement with findings by Chatikobo et al. (2009) who reported that dermatophilosis spread from Matabeleland North, Midlands, and Mashonaland West in that order. During the study by Chatikobo et al. (2009), dermatophilosis had been reported from three provinces, the current study suggests that the disease and its associated tick *A. variegatum* had spread further north-eastwards to include two more provinces, that is, Mashonaland Central and Mashonaland East provinces. Unlimited livestock movement both legal and illegal might have contributed to this spread. According to Estrada-Peña et al. (2008), the southern limit for *A. variegatum* was between latitudes 17°S and 18.5°S. These two facts imply that dermatophilosis and *A. variegatum* have the potential of spreading north-eastwards to new areas in Mashonaland East and Manicaland provinces.

Findings of this study suggest that dermatophilosis occurs throughout the year and that the disease and associated tick *A. variegatum* were spreading from the north-west to the north-eastern parts of Zimbabwe.

## Conclusion

Findings from this study suggest that bovine dermatophilosis was spreading from north-west to the north-east of Zimbabwe; this can be used by the DVS and farmers to plan for the control and management of dermatophilosis through a knowledge of where the disease is most likely to occur next. Interventions that can be instituted may include pre-movement inspection of cattle to ensure that they are not tick infested and that they are clinically disease-free; movement bans should be imposed on clinically affected animals, and these should be treated promptly. Dipping of cattle prior to the movement can aid in reducing the spread of the disease and its associated tick to new areas. A policy of culling previously infected animals that are repeatedly infected should be considered. At destination point, cattle from a dermatophilosis area must be quarantined for at least a month to detect any carriers. The occurrence of space–time clusters indicates areas where more rigorous dermatophilosis control measures should be implemented together with raising stock-owner awareness about the disease. Targeted research should be undertaken to identify the drivers responsible for the spread of dermatophilosis and to determine the socio-economic implications of the disease to the state and livestock keepers.
